# Dialysis adequacy predictions using a machine learning method

**DOI:** 10.1038/s41598-021-94964-1

**Published:** 2021-07-29

**Authors:** Hyung Woo Kim, Seok-Jae Heo, Jae Young Kim, Annie Kim, Chung-Mo Nam, Beom Seok Kim

**Affiliations:** 1 Department of Internal Medicine, Yonsei University College of Medicine, Seoul, Republic of Korea; 2 Department of Biostatistics and Computing, Yonsei University Graduate School, Seoul, Republic of Korea; 3 Department of Internal Medicine, National Health Insurance Service, Ilsan Hospital, Goyangshi, Gyeonggi-do Republic of Korea; 4 Department of Preventive Medicine, Yonsei University College of Medicine, Seoul, Republic of Korea

**Keywords:** Renal replacement therapy, Haemodialysis, Machine learning

## Abstract

Dialysis adequacy is an important survival indicator in patients with chronic hemodialysis. However, there are inconveniences and disadvantages to measuring dialysis adequacy by blood samples. This study used machine learning models to predict dialysis adequacy in chronic hemodialysis patients using repeatedly measured data during hemodialysis. This study included 1333 hemodialysis sessions corresponding to the monthly examination dates of 61 patients. Patient demographics and clinical parameters were continuously measured from the hemodialysis machine; 240 measurements were collected from each hemodialysis session. Machine learning models (random forest and extreme gradient boosting [XGBoost]) and deep learning models (convolutional neural network and gated recurrent unit) were compared with multivariable linear regression models. The mean absolute percentage error (MAPE), root mean square error (RMSE), and Spearman’s rank correlation coefficient (Corr) for each model using fivefold cross-validation were calculated as performance measurements. The XGBoost model had the best performance among all methods (MAPE = 2.500; RMSE = 2.906; Corr = 0.873). The deep learning models with convolutional neural network (MAPE = 2.835; RMSE = 3.125; Corr = 0.833) and gated recurrent unit (MAPE = 2.974; RMSE = 3.230; Corr = 0.824) had similar performances. The linear regression models had the lowest performance (MAPE = 3.284; RMSE = 3.586; Corr = 0.770) compared with other models. Machine learning methods can accurately infer hemodialysis adequacy using continuously measured data from hemodialysis machines.

## Introduction

Dialysis adequacy is an important survival indicator in patients with chronic hemodialysis^1,2^. Recent guidelines recommend that the dialysis dose should be adjusted using a blood test at least once per month and suggest a target single pool Kt/V (spKt/V) of 1.4 per hemodialysis session for patients treated thrice weekly^3^. Although some hemodialysis devices estimate spKt/V using sodium clearance, it is limited to devices from specific manufactures and cannot be applied to all equipment. In contrast, the urea reduction ratio (URR) is easily calculated and used as a standard measurement for the delivered hemodialysis dose^4,5^. However, there are disadvantages; it uses needles, exposes the medical staff and patients to blood, and has costs associated with processing and analyzing blood samples. Additionally, hemodialysis sessions are frequently terminated for reasons such as intradialytic hypotension, vascular access problems, and poor compliance. Therefore, URR is not easily measured regularly in practice.

During hemodialysis, several clinical parameters such as blood flow, ultrafiltration and dialysate flow rates, vessel pressure, temperature, and bicarbonate and sodium levels are continuously generated. Monitoring and recording these parameters in real-time is possible with the commercial software provided with the hemodialysis machine. Considering urea kinetics, some of these measurements, the type of dialyzer, and the dialysis duration may be related to dialysis adequacy. However, the relationship between these measurements and dialysis adequacy is not simple, and models using machine learning (ML) rather than traditional statistical models may be more appropriate for predicting dialysis adequacy. Artificial intelligence has already been used in the healthcare field for medical imaging, natural language processing, and genomics^6^. Recently, studies also used ML or deep learning (DL) (a subfield of ML) to investigate kidney disease^7^.

In this study, we hypothesized that the ML technique could predict dialysis adequacy in chronic hemodialysis patients using clinical demographics and repeated measurements obtained during hemodialysis sessions. This study aimed to build models that predict URR based on repeated measurement data from patients during hemodialysis.

## Results

### Hemodialysis sessions

This study included 1333 hemodialysis sessions corresponding to the monthly examination dates of 61 patients where URR was measured. The mean blood flow was 265.2 mL/min (SD, 41.4), the mean dialysate flow was 571.0 mL/min (SD, 116.3), the mean dialyzer surface area was 1.8 m^2^ (SD, 0.2), the mean URR was 77.7% (SD, 5.3), and the mean total ultrafiltration volume was 2209.0 mL (SD, 826.5) (Table 1). The fivefold cross-validation method divided the data into five approximately equal-sized portions (the minimum and the maximum number of participants was 12 and 13, respectively). The total number of data points was 319,920.

**Table 1:** Study subject characteristics (61 subjects, 1333 sessions).

Characteristics	Mean or n	SD or %
Female	704	53
Age, year	62.9	15.9
Pre-dialysis weight, kg	60.0	12.2
Height, cm	163.8	8.2
Dialyzer surface area, m^2^	1.8	0.2
Pre-dialysis BUN, mg/dL	56.7	16.5
Total ultrafiltration volume, mL	2281.0	826.5
**Type of hemodialysis**		
Conventional HD	670	50
HDF	663	50
**Blood flow rate, mL/min**		
Intercept	265.8	43.7
Coefficient	0.0	0.1
MSE	275.6	591.9
Mean	265.2	41.4
SD	14.3	10.9
**Dialysate flow rate, mL/min**		
Intercept	569.4	118.8
Coefficient	0.0	0.2
MSE	15,134.5	10,652.1
Mean	571.0	116.3
SD	116.0	45.0
**Ultrafiltration volume, mL**		
Intercept	− 4.4	55.0
Coefficient	9.3	3.7
MSE	1312.7	4113.4
Mean	1116.3	458.7
SD	646.6	260.3
Urea reduction ratio (%)	77.7	5.3

*SD* standard deviation, *BUN* blood urea nitrogen, *HD* hemodialysis, *HDF* hemodiafiltration, *MSE* mean square error.

### Model performances

**Figure 1:**
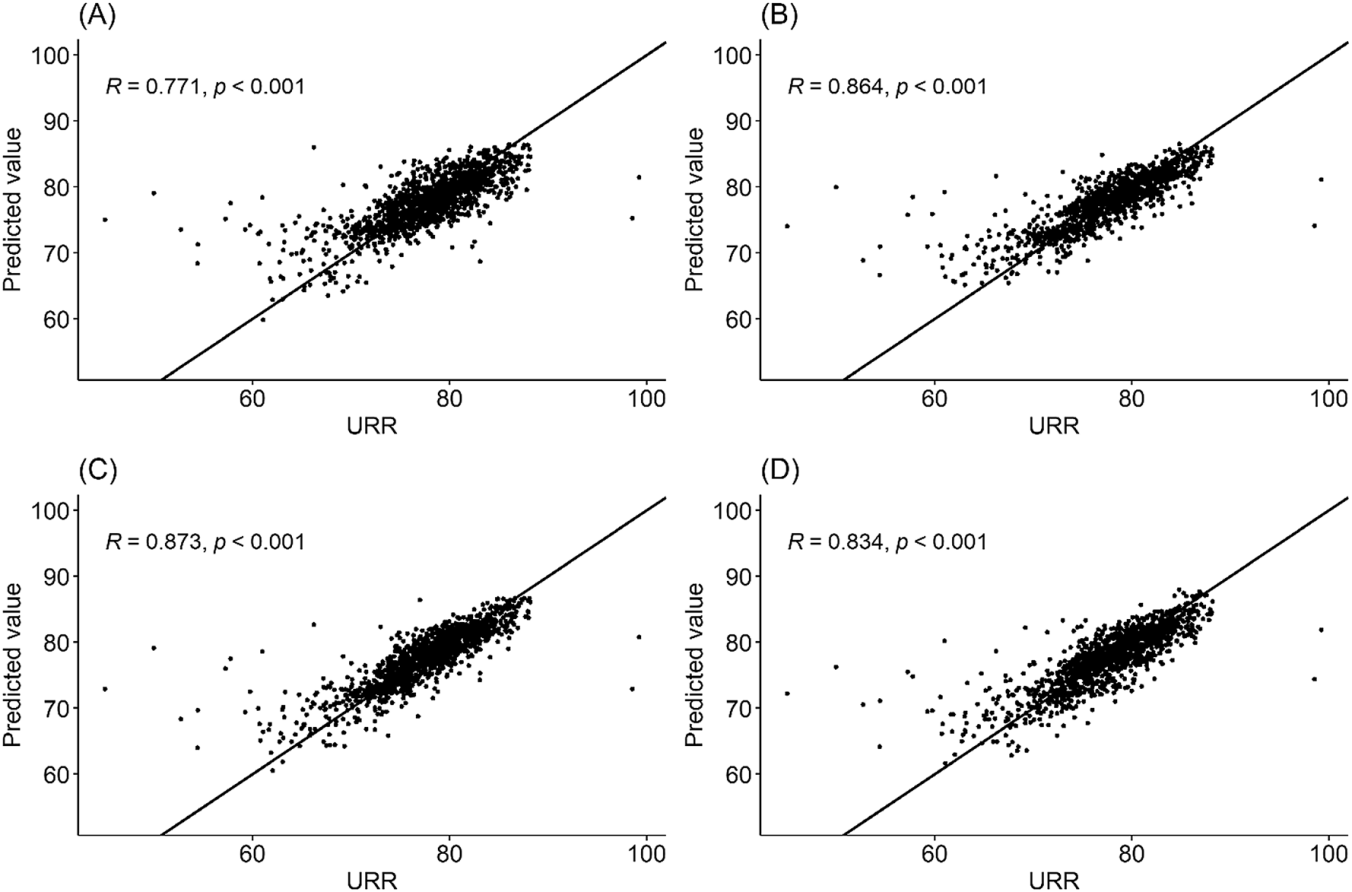
Scatter plots of URR and the predicted value for each model using Spearman’s rank correlation coefficient and the best fit line. (**A**) Linear regression (*R* = 0.771). (**B**) Random forest (*R* = 0.864). (**C**) XGBoost (*R* = 0.873). (**D**) Deep learning with convolutional neural network (*R* = 0.834). *URR* urea reduction ratio, *XGBoost* extreme gradient boosting.

Table 2 summarizes the MAPE, RMSE, and Corr performance measurements for each model using the fivefold cross-validation. For the linear regression model, the models with time-fixed and time-varying covariates had better performances than the model with fixed covariates alone (MAPE = 3.546; RMSE = 3.785; Corr = 0.751). Among the time-varying covariates, the blood flow rate measurement improved performance the most (MAPE = 3.329; RMSE = 3.648; Corr = 0.766). The linear regression model with all covariates had the best performance among the linear regression models (MAPE = 3.284; RMSE = 3.586; Corr = 0.770). However, the linear regression models had a lower performance than the ML and DL models. The ML methods had better performances than the other methods, and the XGBoost model had the best performance among the ML methods (MAPE = 2.500; RMSE = 2.906; Corr = 0.873). The DL models with the convolutional neural network (MAPE = 2.835; RMSE = 3.125; Corr = 0.833) and gated recurrent unit (MAPE = 2.974; RMSE = 3.230; Corr = 0.824) had similar performances. The detailed relationship between URR and the predicted values for each model are depicted using scatter plots in Fig. 1. The results of other hyperparemeter settings are summarized in Supplementary Table S1.

**Table 2:** A performance measurement summary for URR prediction models.

Model	Used covariates	MAPE	RMSE	Corr
Linear regression	Time-fixed covariates^a^	3.546	3.785	0.751
	Time-fixed covariates^a^ + BFR	3.329	3.648	0.766
	Time-fixed covariates^a^ + flow	3.447	3.715	0.757
	Time-fixed covariates^a^ + volume	3.548	3.779	0.753
	All variables	3.284	3.586	0.771
Random forest	All covariates^b^	2.625	3.043	0.864
XGBoost	All covariates^b^	2.500	2.906	0.873
Deep learning with CNN	All covariates^b^	2.873	3.214	0.825
Deep learning with GRU	All covariates^b^	2.857	3.237	0.828

*URR* urea reduction ratio, *MAPE* mean absolute percentage error, *RMSE* root mean square error, *Corr* Spearman’s rank correlation coefficient, *BFR* blood flow rate, *XGBoost* extreme gradient boosting, *CNN* convolutional neural network, *GRU* gated recurrent unit, *BUN* blood urea nitrogen level.

^a^Time-fixed covariates: age, gender, dialyzer surface area, dialysis pre-weight, height, dialysis pre-BUN, and hemodialysis type (conventional hemodialysis or hemodiafiltration). Performance measures were calculated through fivefold cross-validation.

^b^All covariates: time-fixed covariates, BFR, dialysate flow rate, and ultrafiltration volume.

### Feature importance

**Figure 2:**
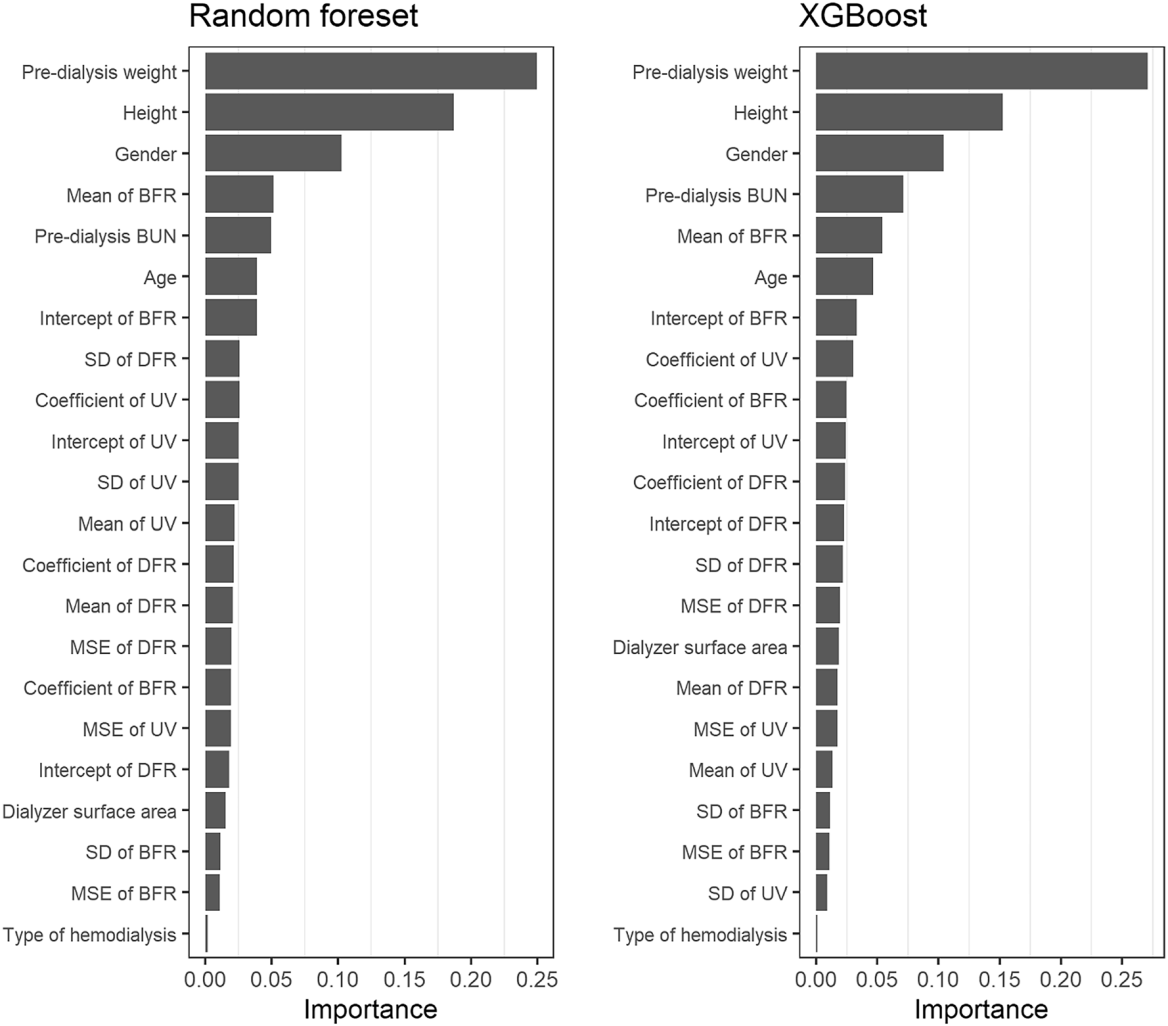
Random forest and XGBoost feature importance plots. *XGBoost* extreme gradient boosting, *MSE* mean squared error, *SD* standard deviation, *BFR* blood flow rate, *DFR* dialysate flow rate, *UV* ultrafiltration volume.

Feature importance was calculated for the random forest and XGBoost models to investigate which covariates affect the URR prediction the most (Fig. 2). Pre-dialysis weight was the most important covariate for predicting URR in both models, followed by height and gender. Artificial features extracted by blood flow rate (i.e., the mean and intercept of the linear regression) had higher importance than other artificial features.

### Sensitivity analyses

Sensitivity analyses were conducted to confirm the fivefold cross-validation results, which were performed in units of sessions instead of patients. After randomizing the sessions, the linear regression, ML, and DL models were trained, and the sensitivity analysis results were similar to the primary results (Table 3). The ML and DL models still performed better than the linear regression model. Sensitivity analysis was also performed on data that eliminated URR outliers to determine how outliers affected model fitting. Sessions with URR values greater than the 95th percentile and less than the 5th percentile were removed. The model performances are summarized in Table 3. The models had better performances after eliminating outliers. However, the performance differences among models were similar before and after outlier removal.

**Table 3:** Sensitivity analysis results for the cross-validation of units as hemodialysis sessions and outlier elimination.

Model	CV in unit of sessions			Outlier elimination		
	MAPE	RMSE	Corr	MAPE	RMSE	Corr
Linear regression	3.248	3.535	0.775	2.673	2.622	0.725
Random forest	2.526	2.944	0.876	2.018	1.996	0.854
XGBoost	2.444	2.854	0.881	1.993	1.968	0.852
Deep learning with CNN	2.891	3.145	0.830	2.220	2.191	0.817
Deep learning with GRU	2.963	3.241	0.826	2.239	2.225	0.811

Models were trained through age, gender, dialyzer surface area, dialysis pre-weight, height, dialysis pre-BUN, hemodialysis type (conventional hemodialysis or hemodiafiltration), and the artificial features of BFR, dialysate flow rate, and ultrafiltration volume.

*CV* cross-validation, *MAPE* mean absolute percentage error, *RMSE* root mean square error, *Corr* Spearman’s rank correlation coefficient, *XGBoost* extreme gradient boosting, *CNN* convolutional neural network, *GRU* gated recurrent unit, *BUN* blood urea nitrogen level.

## Discussion

Current guidelines recommend checking dialysis adequacy once per month because dialysis adequacy is related to the prognosis of end-stage kidney disease patients^3^. However, determining adequacy is challenging owing to the cost and blood exposure. The prediction model used parameters that determine hemodialysis efficiency, such as blood flow and dialysate flow rates, dialysis time, and the dialyzer type^8–10^. However, it is difficult to predict dialysis adequacy using these parameters through traditional statistical methods as the relationships between these parameters and urea clearance are not linear; they frequently change during hemodialysis with fluctuations in blood pressure or other symptoms. This study showed that ML and DL models using continuous measurements obtained during hemodialysis predicted dialysis adequacy. Furthermore, there are significant implications in repeated measurements from hemodialysis machines for making such predictions. For example, there is no additional cost because the adequacy predictions are based on measurements obtained from any hemodialysis machine, making this approach useful when remote monitoring is required, such as with at-home hemodialysis.

DL has been mainly used for image processing, although recently, DL has also been used for predicting laboratory results or the short-term prognosis of patients based on continuously measured data. Additionally, large-scale intensive care unit datasets, such as the Medical Information Mart for Intensive Care III^11^ and eICU Collaborative Research Database^12^, and intra- or post-operative vital sign data are now available for use in research^13^. Various studies have also used DL to investigate hemodialysis. Akl et al.^14^ suggested decades ago that the neural network can achieve artificial-intelligent dialysis control, and studies on intradialytic hypotension predictions^15–18^, the optimal dry weight setting^19^, and anemia control^20^ for hemodialysis have been presented. DL in research has also expanded to other kidney diseases to predict acute kidney injury outcomes^21,22^ and hyperkalemia^23^. Despite challenges, such as data cleansing costs, the required modeling resources, and algorithm validations, the DL approach is expected to improve the prognosis of hemodialysis patients in the future.

There are some limitations to our study. First, despite a relatively large number of hemodialysis sessions, this study was conducted on a small number of patients. For this reason, DL models might show lower performances than random forest or XGBoost models in this study. A large, prospective study is needed to validate our model. Second, some factors influencing the blood urea nitrogen level during hemodialysis were not considered (e.g., the catabolic status, the exact residual renal function, and access recirculation). However, this study was based on outpatient clinic data with few acutely ill patients, and ultrafiltration (a factor affecting the blood urea nitrogen level) was included in our model. Therefore, the effect of the catabolic status was minimized. Finally, URR has been used as a standard method to measure the hemodialysis dose^4^. However, the current guidelines do not recommend using URR for hemodialysis adequacy. Nevertheless, URR is widely used in clinics because it is easy to calculate and has a similar sensitivity to urea reduction compared with other methods^24^. Models that predict spKt/V require verification in the future.

In conclusion, ML can accurately infer hemodialysis adequacy through repeatedly measured data during hemodialysis sessions. We expect to be able to develop personalized hemodialysis profile recommendation models through prospective data collection soon.

## Materials and methods

### Study population

The data were extracted from the Severance Hospital hemodialysis database, which stores information about each hemodialysis session. A total 21,004 sessions of 75 outpatients aged over 19 which were automatically recorded in the Therapy Data Management System from May 2015 to September 2020 were screened. Among them, 61 patients who were examined for dialysis adequacy regularly were finally selected and clinical information including dialysis adequacy was additionally collected. The study was performed following the Declaration of Helsinki principles, and the Severance Hospital institutional review board approved this study (no. 4-2021-0056) and waived informed consent as only de-identified, previously collected data was accessed.

### Data collection and measurements

Demographic and anthropometric data (including sex and age) were collected corresponding to the hemodialysis date from electric medical records. Blood pressure, the vascular access type (arteriovenous fistula, arteriovenous graft, and catheter), and the dialyzer type (surface area) were recorded at the initiation of each hemodialysis session. Data, including blood flow and ultrafiltration rates, bicarbonate and sodium levels, dialysate flow, vein and artery pressures, and the dialysate temperature, were measured every minute from the start of each session unless problems or interventions occurred. Monitoring software linked to each dialysis machine recorded the hemodialysis measurements in real-time and collected 240 measurements (about 4 h) from each session; missing values were completed using an interpolation method. URR (the blood urea concentration decrease [%] during hemodialysis) was measured as an indicator of dialysis adequacy. All hemodialysis sessions included in this study used the Fresenius 5008S (Fresenius Medical Care, Bad Homburg, Germany) hemodialysis device.

### Model building

Linear regression was considered the base model for performance comparisons with ML and DL algorithms. Random forest^25^ and XGBoost^26^ were chosen for the ML algorithms. The convolutional neural network and gated recurrent unit^27^ architectures were chosen for the DL algorithms to extract features from time-varying covariates. The DL algorithms were trained with a batch size of 128, Adam optimizer^28^ and the root mean squared error (RMSE) loss function. The detailed architectures of the DL algorithms are illustrated in Supplementary Figure S1 and Supplementary Figure S2. The hyperparameters were optimized to minimize the RMSE through a random search with fivefold cross-validation in ML algorithms. All selected hyperparameters are described in Supplementary Table S1.

Covariates were normalized to have values between 0 and 1 in the DL algorithms, which can automatically extract features from time-varying covariates. In contrast, the linear regression and ML algorithms require a data pre-processing step to extract artificial features from time-varying covariates. Thus, the means and standard deviations (SDs) from time-varying covariates by session for the linear regression and ML algorithms were extracted, and then, a linear regression for the time-varying covariates by session was implemented. From this linear regression, the intercept, coefficient, and mean squared error (MSE) were extracted.

### Statistical analyses

Descriptive statistics were used to describe covariates. Categorical variables were expressed as the number of patients and percentages, and continuous variables were presented as the mean and SD. The mean absolute percentage error (MAPE), RMSE, and Spearman’s rank correlation coefficient (Corr) were calculated for performance evaluation using fivefold cross-validation. Two sensitivity analyses were also performed for result confirmation. First, each session was regarded as belonging to a different person. Then, the main analysis was repeated after eliminating URR outliers. Analyses, including the linear regression and ML algorithms, were performed using R software (version 3.6.1; www.r-project.org; R Foundation for Statistical Computing, Vienna) with the authors’ own program written code using the XGBoost and ranger packages. Python software (version 3.7; www.python.org; Python Software Foundation, Wilmington) was used with the Keras library for the DL algorithms. A computer with a Xeon processor (24 core, Intel, USA) and Quadro RTX 6000 (Nvidia, USA) was used for all analyses.

## Supplementary Information

Supplementary Information.

## Data Availability

The datasets generated during the current study are not publicly available due to the data security requirement of our hospital.
