# Supplementary material for: Dialysis adequacy predictions using a machine learning method
Source: Sci Rep. 2021 Jul 29;11:15417. doi: 10.1038/s41598-021-94964-1 (PMC8322325; doi:10.1038/s41598-021-94964-1)
Supplement: Supplementary file 1 — Supplementary Information. [file 41598_2021_94964_MOESM1_ESM.docx]

**Supplementary Materials**

**Dialysis Adequacy Predictions Using a Machine Learning Method**

Corresponding Author: Beom Seok Kim MD, PhD (docbsk@yuhs.ac) and Chung-Mo Nam MD, PhD (cmnam@yuhs.ac)

**Table of contents**

***Supplementary Table S1.***A performance measurement summary for URR prediction models
for various hyperparameter settings.

***Supplementary Table S2.***Summary of hyperparameters for random forest and XGBoost.

***Supplementary Figure. S1****.* Schemas of CNN model

***Supplementary Figsure S2****.* Schemas of GRU model

***Supplementary Table S1.***A performance measurement summary for URR prediction models
for various hyperparameter settings.

| Model | MAPE | RMSE | Corr |
| --- | --- | --- | --- |
| Random Forest with 2 mtry | 2.832 | 3.190 | 0.849 |
| Random Forest with 4 mtry | 2.625 | 3.043 | 0.864 |
| Random Forest with 8 mtry | 2.647 | 3.089 | 0.863 |
| XGBoost with 2 max_depth | 2.608 | 2.950 | 0.860 |
| XGBoost with 4 max_depth | 2.500 | 2.906 | 0.873 |
| XGBoost with 8 max_depth | 2.526 | 2.936 | 0.872 |
| CNN with 8 filters | 3.113 | 3.331 | 0.805 |
| CNN with 16 filters | 2.873 | 3.214 | 0.825 |
| CNN with 32 filters | 3.262 | 3.512 | 0.782 |
| GRU with 8 memory cells | 2.887 | 3.241 | 0.830 |
| GRU with 16 memory cells | 2.857 | 3.237 | 0.828 |
| GRU with 32 memory cells | 2.980 | 3.261 | 0.818 |
| LSTM with 8 memory cells | 2.894 | 3.290 | 0.831 |
| LSTM with 16 memory cells | 2.949 | 3.271 | 0.820 |
| LSTM with 32 memory cells | 3.032 | 3.313 | 0.814 |

***Abbreviations:*** mtry, number of variables to possibly split at in each node; max_depth, maximum depth of a tree; CNN, convolutional neural network; GRU, gated recurrent unit; LSTM, long short-term memory.

***Notes:*** We only considered a hyperparameter that impact performance most for each model.

***Supplementary Table S2.***Summary of hyperparameters for random forest and XGBoost.

| Model | Hyperparameter | Description | Value |
| --- | --- | --- | --- |
| Random Forest | mtry | Number of variables to possibly split at in each node | 4 |
|  | num.trees | Number of trees | 300 |
| XGBoost | subsample | Subsample ratio of the training instances | 0.8 |
|  | colsample_bytree | Subsample ratio of columns when constructing each tree | 0.8 |
|  | alpha | L1 regularization term on weights | 0 |
|  | lambda | L2 regularization term on weights | 0.5 |
|  | max_depth | Maximum depth of a tree | 4 |
|  | eta | Step size shrinkage used in update to prevents overfitting | 0.01 |

***Abbreviations:*** XGBoost, extreme gradient boosting.

***Supplementary Figure S1****.* Schemas of CNN model


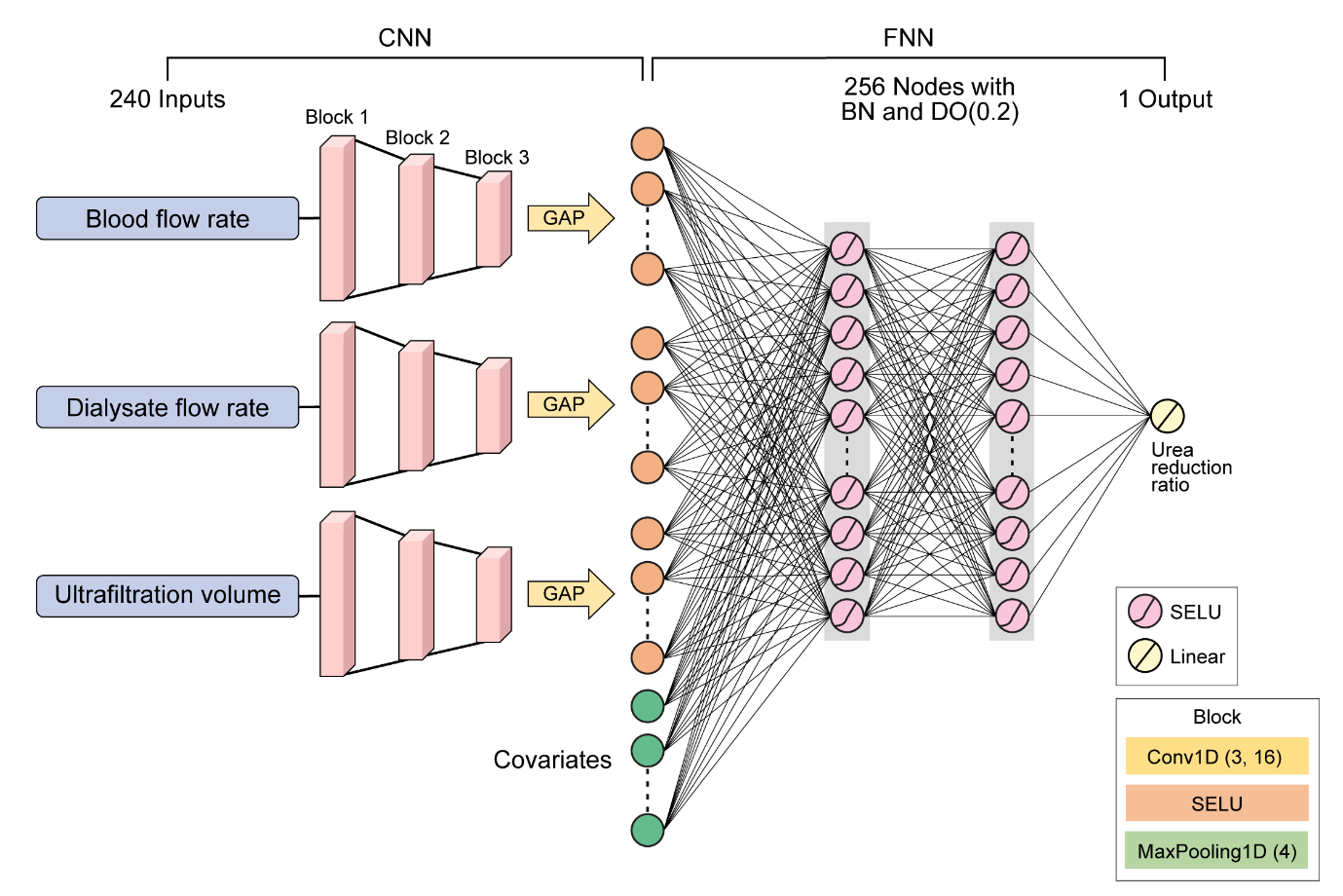


***Abbreviations:*** CNN, convolutional neural network; FNN, feedforward neural network; GAP, global average pooling; BN, batch normalization; DO, dropout; SELU, scaled exponential linear unit.

***Notes:*** DO (dropout rate), Conv1D (kernel size, number of filters), MaxPooling1D (pooling window size)

***Supplementary Figure S2****.* Schemas of GRU model


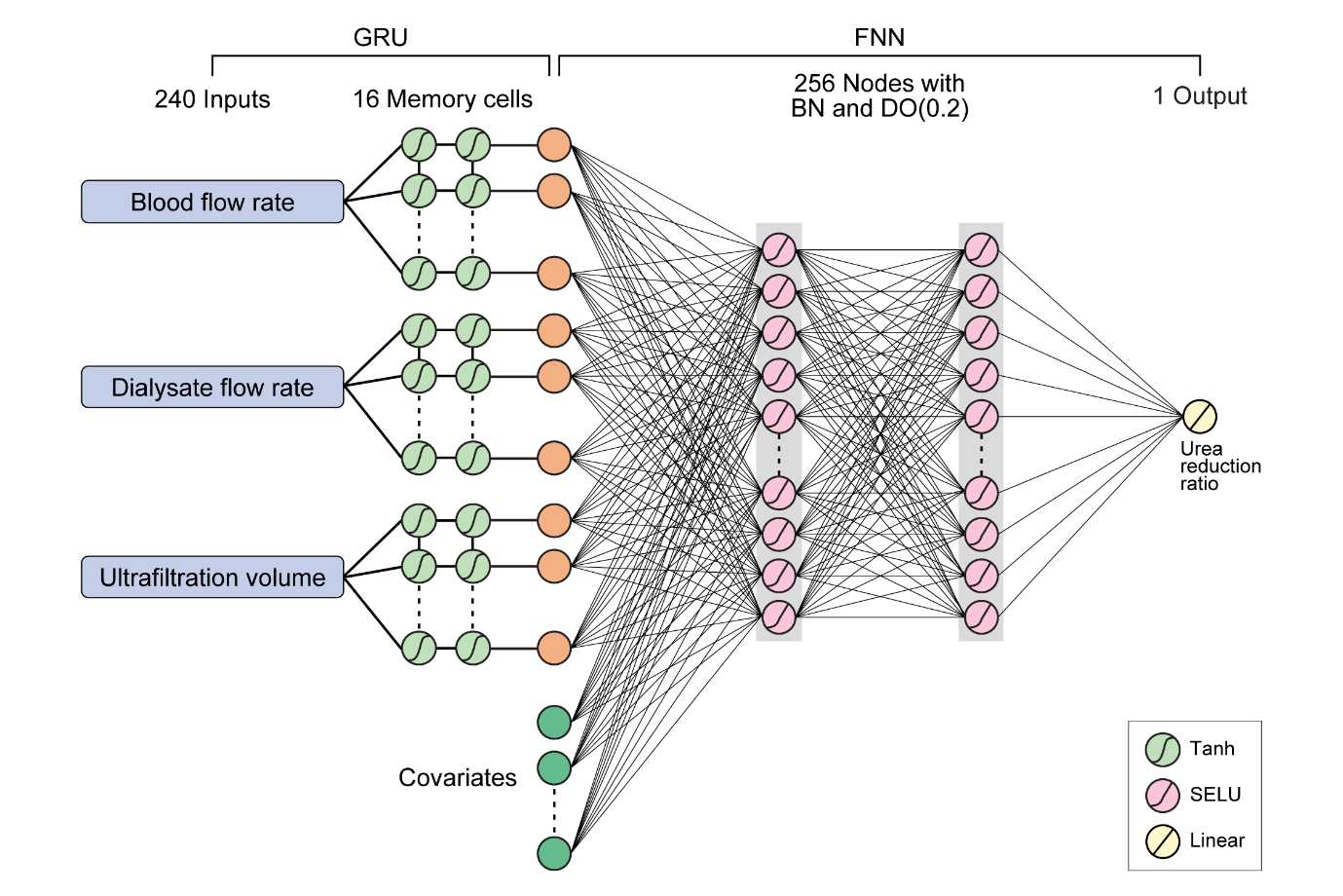


***Abbreviations:*** GRU, gated recurrent unit; FNN, feedforward neural network; BN, batch normalization; DO, dropout; Tanh, hyperbolic tangent; SELU, scaled exponential linear unit.

***Notes:*** DO (dropout rate), GRU with 240 time steps was used.
